# Why Cells and Viruses Cannot Survive without an ESCRT

**DOI:** 10.3390/cells10030483

**Published:** 2021-02-24

**Authors:** Arianna Calistri, Alberto Reale, Giorgio Palù, Cristina Parolin

**Affiliations:** Department of Molecular Medicine, University of Padua, 35121 Padua, Italy; alberto.reale@studenti.unipd.it (A.R.); giorgio.palu@unipd.it (G.P.); cristina.parolin@unipd.it (C.P.)

**Keywords:** ESCRT, viruses, cellular membranes, extracellular vesicles, HSV-1

## Abstract

Intracellular organelles enwrapped in membranes along with a complex network of vesicles trafficking in, out and inside the cellular environment are one of the main features of eukaryotic cells. Given their central role in cell life, compartmentalization and mechanisms allowing their maintenance despite continuous crosstalk among different organelles have been deeply investigated over the past years. Here, we review the multiple functions exerted by the endosomal sorting complex required for transport (ESCRT) machinery in driving membrane remodeling and fission, as well as in repairing physiological and pathological membrane damages. In this way, ESCRT machinery enables different fundamental cellular processes, such as cell cytokinesis, biogenesis of organelles and vesicles, maintenance of nuclear–cytoplasmic compartmentalization, endolysosomal activity. Furthermore, we discuss some examples of how viruses, as obligate intracellular parasites, have evolved to hijack the ESCRT machinery or part of it to execute/optimize their replication cycle/infection. A special emphasis is given to the herpes simplex virus type 1 (HSV-1) interaction with the ESCRT proteins, considering the peculiarities of this interplay and the need for HSV-1 to cross both the nuclear-cytoplasmic and the cytoplasmic-extracellular environment compartmentalization to egress from infected cells.

## 1. Introduction

Membrane-surrounded organelles characterize eukaryotic cells and guarantee the compartmentalization of distinctive processes and functions. Intracellular membranes not only maintain the integrity of these compartments but, thanks to finely tuned vesicle trafficking, also play a pivotal role in the crosstalk between organelles themselves. Dynamic, constant and controlled remodeling processes enable the exchange of signals, information and materials between membranes that is crucial for the functioning of biological systems [1]. Two main types of membrane involutions can be formed. The first type of vesicles excludes the cytosolic environment and occurs during “classical” budding events, such as endocytosis. The second type of vesicles, instead, originates from a “reverse-topology” membrane scission which includes the cytosol and is mediated by the endosomal sorting complex required for transport (ESCRT) machinery [2]. This network of proteins is involved in different essential cellular processes, such as cytokinesis, autophagy, multivesicular body (MVB) and extracellular vesicle (EV) biogenesis, plasma, nuclear and endolysosomal membrane repair [2,3]. This list is far from being exhaustive, but represents the pathways involving ESCRTs that are better characterized. Viruses, being obligate intracellular parasites, have evolved to hijack highly conserved cellular pathways throughout their replication cycle [4]. Thus, and not surprisingly, viruses exploit the host intracellular membrane trafficking machinery to execute crucial steps of infection, such as (i) entering the target cell; (ii) transporting their genomic materials to the site of replication; (iii) if enveloped, acquiring their external lipid coating; (iv) exiting from infected cells. At the same time, several enveloped and non-enveloped viruses induce profound membrane remodeling/proliferation in infected cells to create specialized compartments where viral genome replication and/or new virion assembly occurs [5,6,7]. Interestingly, some insect and plant viruses are able to modify mitochondrial and peroxisomal membranes for their replication [7]. Finally, EVs play a broad-spectrum role in the pathogenesis of viral infection. Indeed, viruses not only adopt exosomes to accomplish specific steps of their life cycle, but also exploit these EVs to transfer both viral and cellular factors, such as proteins and non-coding RNAs, outside the infected cells to promote infection and to escape from the immune system [8].

Here, we review the similarities and differences of various ESCRT-dependent cellular processes, including EV biogenesis, emphasizing mechanisms of ESCRT recruitment by viruses. Furthermore, we focus on how the herpes simplex virus type 1 (HSV-1), a complex DNA-enveloped virus, interacts with ESCRT proteins to cross the nuclear envelope and egress from infected cells.

## 2. The ESCRT Machinery: An Overview

The ESCRT machinery and its associated factors include a network of different proteins (roughly 20 in yeast and 30 in mammals) that are sequentially recruited to the inner surface of the membrane necks of vesicles, mostly budding away from the cytosol (the so-called “reverse topology” budding event). ESCRT proteins were originally identified in budding yeasts in studies aimed at the identification of factors involved in the biogenesis of the MVBs [9,10]. MVBs contain intraluminal vesicles (ILVs) that arise from the budding of the limiting endosomal membrane into the lumen of the organelle. When MVBs fuse to the lysosomes, the content of those ILVs is degraded [11]. This mechanism of degradation has been well described in the case of both misfolded cell surface proteins and of G-coupled proteins and tyrosine kinase receptors, channels and transporters, which need to be downregulated after responding to specific stimuli [12]. The nascent ILVs are connected to the limiting membrane by a narrow membrane neck, which must be cut to release them into the lumen. In MVB biogenesis, the ESCRT machinery drives both budding and scission of ILVs. In different pathways, proteins other than the ESCRTs are responsible for the formation of the vesicle/membrane neck, while ESCRTs and associated factors drive membrane scission. Although ESCRT machinery is the master player of reverse topology budding events, some exceptions to this rule are reported in the literature. For instance, in the peroxisome biogenesis of budding yeasts, one of the ESCRTs, namely ESCRT-III, which is described in the next paragraph, is involved in a classical topology membrane scission event. Indeed, it allows the scission of pre-peroxisomal vesicles from the endoplasmic reticulum (ER) membrane into the cytosol [13].

**Figure 1:**
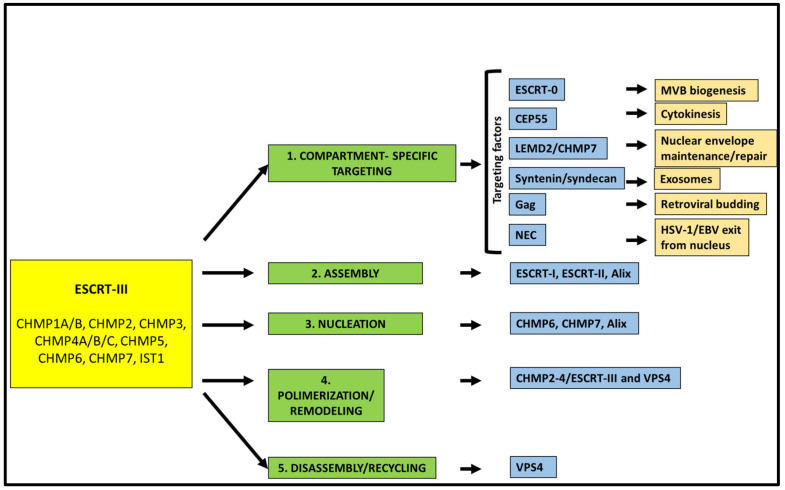
Schematic representation of the sequential steps leading to ESCRT-III polymerization and functioning. The main factors involved in each step are reported, along with the biological processes in which ESCRT-III plays a role based on its compartment-specific recruitment (light orange rectangles).

The core of the ESCRT machinery consists of three complexes (ESCRT-I, ESCRT-II, ESCRT-III), as well as of the associated protein Alix (BRO1 in yeast) and the AAA-type ATP-ase, the vacuolar protein sorting (VPS) 4 [2,3,10]. ESCRT-I is a stalk-shaped heterotetrameric complex of proteins encompassing tumor suppressor gene 101, TSG101 (Vps23 in yeast), VPS28, VPS37 (from A to D), and UBAP1, MVB12A, or MVB12B [14,15,16]. ESCRT-II is a Y-shaped complex constituted by VPS22 (also known as EAP30), VPS36 (also known as EAP45), and two copies of VPS25 (also known as EAP20) [17]. ESCRT-III is formed by the so-called charged multivesicular body (CHMP) proteins 1 to 7, i.e., CHMP1A/B (Did2 in yeast); CHMP2A/B (Vps2 in yeast); CHMP3, (Vps24 in yeast); CHMP4A/B/C (Snf7 in yeast); CHMP5 (Vps60 in yeast); CHMP6 (Vps20 in yeast) and CHMP7 (Chm7 in yeast). Finally, increased sodium tolerance-1 (IST1) is also part of this complex [18]. While ESCRT-III is directly involved in remodeling and severing membranes, ESCRT-I and ESCRT-II often function together as assembly factors for ESCRT-III. Indeed, those two complexes cooperate to bring ESCRT-III to the site of the membranes where budding events are going to take place [19]. On the other hand, Alix [20,21] can work as an alternative way for the recruitment and activation of ESCRT-III. Indeed, Alix, via its Bro1 domain [22], can directly bind CHMP4. In some cases, the His domain-containing protein tyrosine phosphatase HD-PTP, an Alix-like protein also bearing a Bro1 domain, can replace Alix itself in recruiting CHMP4 [23,24]. An additional Bro1 containing protein, Bro1 domain/CAAX motif containing-protein (BROX), has been suggested to function in an Alix-like manner [25]. ESCRT-III assembly at the side of vesicle budding and membrane pinching off are dynamic processes. The AAA+ ATPase VPS4 [26] has a crucial role in recycling ESCRT-III subunits by extracting ESCRT-III monomers from the assembly. VPS4 functions as a hexamer and is characterized by an N-terminal microtubule interaction and transport (MIT) domain [27], along with a catalytic domain [28]. The MIT domain can bind to the MIT-interaction motifs (MIMs) that are present within ESCRT-III components. In particular, VPS4 has been shown to strongly bind to the MIM containing ESCRT-III proteins CHMP1, CHMP2, CHMP6, and IST1 [29,30]. ESCRT-III and VPS4 are universally involved in ESCRT-dependent membrane dynamic processes [31] that are reviewed in the following sections. Thus, it is not surprising that both ESCRT-III and VPS4 have homologs in archaea [32,33,34,35,36]. At the basis of most of the reverse topology fission events, indeed, are sequential steps involving ESCRT-III (Figure 1).

First, ESCRT-III is targeted to the correct membrane/cell compartment. In most cases, this occurs thanks to proteins that act as compartment-specific targeting factors by forming molecular bridges for the recruitment of the ESCRT machinery (Figure 1). Several of these factors have been characterized over the years, including the cellular and viral proteins ESCRT-0, the centrosomal protein 55 (CEP55), syndecan/syntenin, Gag, LEMD2 (LEM Domain 2 protein)–CHMP7, BFRF1 and UL31/34. Each of these proteins contributes to the targeting of the ESCRT machinery towards a certain cellular compartment and facilitates a specific function of this complex. For instance, and as better detailed below, syndecan/syntenin are involved in the biogenesis of exosomes, CEP55 in the ESCRT-III mediated cell abscission during cytokinesis, BFRF1 and UL31/34 enable the egress of two herpesviruses, Epstein–Barr virus (EBV) and the herpes simplex virus type 1 (HSV-1), respectively, from the inner nuclear membrane (INM) of infected cells. Usually, ESCRT-III does not directly interact with these proteins, but it needs the involvement of assembly factors as the above-mentioned ESCRT-I/ESCRT-II, Alix and HD-PTP. In particular, Alix and HD-PTP, by binding both ESCRT-III and ESCRT-I [37,38], function as ESCRT-II alternatives. After assembly, nucleation and polymerization of ESCRT-III take place. In these steps, ESCRT-III components, and in particular CHMP4, need to be activated, a crucial process that can be mediated by both ESCRT-I/ESCRT-II or ESCRT-0/Alix [39,40]. ESCRT-III function relies on a finely tuned balance between the upstream factors that allow filament nucleation and the downstream proteins, including VPS4, that control filament polymerization rate [10]. If this balance is altered, the consequences for the cell can be detrimental. CHMP4-containing ESCRT filaments, as well as filaments composed of other ESCRT-III proteins (i.e., CHMP-2 and CHMP-3), are highly dynamic, a feature essential to determine membrane remodeling and scission. Furthermore, CHMP4 interacts with CHMP3 leading to the sliding of polymers that contribute to the ability of ESCRT-III filaments to change their architecture to adapt to the different phases of the membrane fission [41]. Interestingly, there is evidence that also VPS4 plays a role during the ESCRT-III remodeling process, in addition to functioning as a recycling factor for the proteins composing this complex [42].

## 3. ESCRT Machinery Functions in Fundamental Cellular Pathways

**Figure 2:**
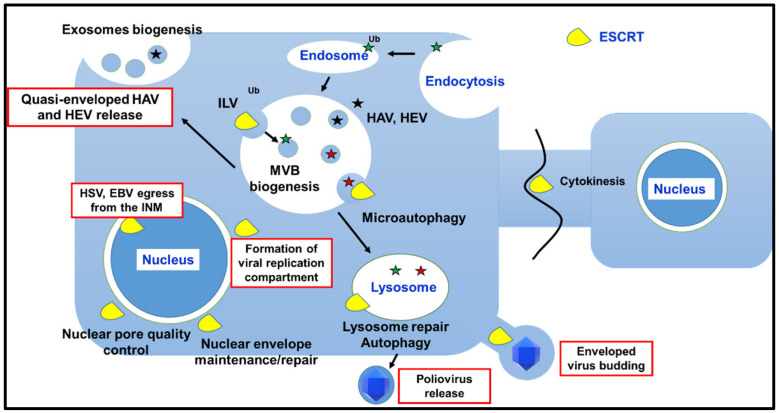
Schematic representation of the better-characterized cellular and viral processes (highlighted by red rectangles) that involve ESCRT-machinery or specific ESCRT factors. HAV stands for hepatitis A virus; HEV for hepatitis E virus; HSV for herpes simplex virus; EBV for Epstein–Barr virus; INM for inner nuclear membrane. The involvement of ESCRT machinery in the viral replication cycles is discussed in the next paragraphs.

Membrane remodeling followed by reverse topology budding events and/or fission enables different fundamental cellular processes, including biogenesis of MVBs; cytokinesis; plasma and intracellular membrane maintenance/repair/reformation; micro- and macroautophagy. The involvement of the ESCRT machinery in these pathways, with a focus on results obtained in mammalian cells, is discussed in the next paragraphs and summarized in Figure 2.

### 3.1. ESCRTs Involvement in MVB Biogenesis

Historically, one of the first studied functions of ESCRTs was their central role in the biogenesis of MVBs. Typically, MVBs are involved in protein trafficking throughout the endosomal-lysosomal pathway [43]. Transmembrane proteins endocytosed from the plasma membrane face two main fates: (i) they can be recycled back to the plasma membrane or the Golgi apparatus (GA) or can be retained to the limiting membrane of the MVB itself; (ii) they can be targeted to the ILVs and thus degraded, once mature MVBs fuse with the lysosomes. The ESCRT machinery is involved in both protein sorting as well as in ILV biogenesis and budding. MVBs display other important biological functions mostly related to the release of EVs, which can significantly influence intercellular signaling as well as the extracellular microenvironment [44,45,46]. Indeed, ILVs can give rise to exosomes upon a fusion of MVBs with the plasma membrane [43,47]. In conclusion, intracellular trafficking of MVBs, which is mainly regulated by Rab proteins, follows at least three different pathways: degradation (fusion with lysosomes), back-fusion (recycling of proteins) and secretion (release of exosomes). Central to all is the biogenesis of ILVs, which begins in early endosomes [48,49]. Proteins intended for degradation are usually marked by ubiquitin. Ubiquitin is a highly conserved protein of 76 amino acids that can be covalently linked to target proteins through a multistep process known as ubiquitylation [50]. Ubiquitin plays a central role in the MVB biogenesis pathway. Indeed, mono-ubiquitylation is necessary and sufficient to trigger the ESCRT-dependent endosomal sorting of membrane proteins and their degradation through the MVB/lysosomal pathway [51,52]. Moreover, ubiquitin plays a role in the recruitment and function of ESCRT components during the ILVs formation. Endosomal proteins, typically marked by lysine-63-linked ubiquitin, enter into the nascent ILVs by interacting with a ubiquitin-binding complex, known as ESCRT-0, constituted by HRS and STAM (Vps27 and Hse1 in budding yeast, respectively) [2,53]. ESCRT-0 is recruited to the early endosome limiting membrane by the interaction of an HRS FYVE domain with phosphatidylinositol 3-phosphate [54,55], a lipid that enriches this type of membrane. Once assembled as a heterotetrameric complex (two HRS and two STAM subunits) [56], ESCRT-0 can efficiently bind ubiquitylated cargoes since it displays several ubiquitin-binding domains (UBDs). Clathrin, along with ESCRT-0, concentrates ubiquitylated proteins in specific patches where sorted cargoes are handed over to ESCRT-I, once again thanks to ubiquitin recognition. Indeed, TSG101 contains a ubiquitin E2 variant (UEV) domain that binds ubiquitin. Furthermore, the mutually exclusive ESCRT-I components MVB12 or UBAP1 can bind ubiquitin through the ubiquitin associated (UBA) or the solenoid of overlapping UBA (SOUBA) domains, respectively. ESCRT-I then binds ESCRT-II, which contains a GRAM-like ubiquitin-binding in Eap45 (GLUE) domain. Finally, ESCRT-III and VPS4 are recruited to the sites of the endosomal membranes where cargo proteins have been concentrated, leading to membrane deformation, ILVs formation and budding [42,57,58,59]. As protein ubiquitylation is crucial for ESCRT-mediated cargo sorting into ILVs, and ESCRT components themselves (i.e., HRS, TSG101 and Alix) can be ubiquitylated, it is not surprising that both ubiquitin ligases and de-ubiquitylating enzymes are associated with the ESCRT machinery, with a key regulative role [60]. Of note, ESCRT-III has no known UBDs, and cargo deubiquitylation, at least in budding yeast, takes place prior to ILVs sorting, a process that contributes to maintaining the correct cytosolic levels of free ubiquitin (Figure 2). Finally, in mammalian cells, ILVs biogenesis can occur independently from ESCRT-0 and ESCRT-I. In this alternative pathway, Alix binds and sorts cargo proteins irrespectively of their ubiquitylation state [61,62,63]. Interestingly, the proteoglycan syndecan can recruit the ESCRT machinery via Alix along with syntenin-1, which functions as a bridge between the first two factors [64]. The syndecan-syntenin-Alix complex is, then, involved in the engagement of ESCRT-III during the biogenesis of exosomes. Exosomes are the smallest subclass of EVs with a diameter ranging from 30 to 120 nm. In addition to the Alix-mediated mechanisms, also classical ESCRT-I/ESCRT-II-dependent engagement of ESCRT-III has been described in exosome formation, as well as ESCRT machinery-independent processes [8]. Exosomes play a key role in cell-to-cell signaling and are emerging as important tools for diagnosis/treatment of diseases, cancer included, and as prognostics markers. Indeed, their cargo, which encompasses not only proteins but also lipids and nucleic acids, such as micro-ribonucleic acids (miRNAs) and long non-coding RNAs (lncRNAs), as well as functional messenger RNAs (mRNAs), is protected and can be delivered to target cells or quantified upon vesicle harvesting [8].

### 3.2. Role of ESCRT Machinery in Autophagy

Taking into account its role in the endolysosomal pathway, the involvement of the ESCRT machinery in autophagy is not unexpected. Indeed, autophagy is a process central for cell survival, as, among the best characterized of its functions, it mediates degradation of large intracellular materials (as organelles) by lysosomes. The autophagosome is a double-membrane structure that can originate from a variety of cellular membranes, including the ER, mitochondria and plasma membrane [65]. Autophagy is divided into macroautophagy and microautophagy. In the first case, cytoplasmic content is surrounded by a double membrane phagosome that can then fuse with lysosomes to degrade intraluminal substances. Macroautophagy plays a crucial role not only for cell catabolism, but also as a defense against intracellular pathogens and for removing damaged organelles. Microautophagy, instead, is based on the budding of ILVs from the endosome/lysosome membranes for degradation of specific cytosolic cargoes. In this sense, although the process closely resembles the MVB biogenesis, microautophagy is a distinct pathway with respect to MVB formation, as it does not involve endocytosed proteins but cytosolic molecules. ESCRT machinery plays a role in both types of autophagy [66,67,68]. This is not surprising in the case of microautophagy due to the aforementioned similarities. However, ESCRT-III and VPS4 are also recruited to the forming autophagosome, where they participate in the sealing of the double membrane coating. Furthermore, when ESCRT functions are blocked, cells accumulate autophagosomes, thus suggesting that ESCRTs are also part of the machinery allowing the fusion of autophagosomes with lysosomes [69,70].

### 3.3. Role of the ESCRT Machinery in Cytokinesis

Over the years, different studies carried on both in vitro and in model organisms (i.e., the fruit fly Drosophila melanogaster and the nematode Caenorhabditis elegans) have revealed the involvement of specific ESCRT factors also in the final phases of cell cytokinesis, the process is known as abscission [71,72,73,74,75]. Specifically, TSG101 and Alix play a pivotal role in this context, as they are directly engaged to the midbody ring by interactions with the centrosomal protein of 55 kDa (CEP55) [71,72,74,76,77]. Furthermore, TSG101 binds to septin 9, one of the septin ring proteins [78]. TSG101 and Alix work in parallel, allowing ESCRT-III nucleation and assembly at the midbody ring [79,80]. ESCRT-III is bound to the membrane via the microtubule interacting and trafficking domain containing 1 (MITD1) protein [81,82] and polymerizes in filaments that stretch out like arms from each side of the midbody ring. VPS4 functions by continuously remodeling these arms [42,58,83]. In the late phases of abscission, part of ESCRT-III and VPS4 localizes at the level of a secondary and thinner intracellular bridge between daughter cells that is generated by actin cytoskeleton remodeling along with microtubule reorganization and fusion of endocytic vesicles to the plasma membrane. ESCRT-III recruits the AAA-ATPase spastin that determines microtubule severing, followed by cell scission [75,84,85,86]. Interestingly, ESCRT-III and VPS4 homologs have also been characterized in archaea [35,87], where they work in concert with the cell division machinery (Cdv) to regulate abscission. Thus, it is likely that ESCRT-III/VPS4 involvement in the late phases of cytokinesis represents the ancestral role of the ESCRT machinery. Finally, the ESCRT-III function is modulated by abscission checkpoint regulators through different mechanisms [73,88,89]. In particular, CHMP4C plays a crucial role in this context. Indeed, it has been recently shown that a CHMP4C polymorphism (CHMP4CA232T), which disrupts the abscission checkpoint, leads to genome instability and is associated with different types of cancer [90]. On the other hand, and not surprisingly, failures in general of the process of abscission are connected with tumorigenesis [91]. This finding suggests that ESCRT-III components may be connected to oncogenesis via the induction of non-checkpointed aneuploidy. On the other hand, and not surprisingly, failures in the process of abscission, by leading to cells with an unstable tetraploid content, are, in general, connected with tumorigenesis [91]. Under this respect, different roles in cancer development are emerging for CEP55 [92]. Furthermore, it has been reported that when full chromosomes or part of them do not properly segregate, they can recruit their own nuclear membrane and form micronuclei (MN). Importantly, due to protein distribution defects within the inner membrane, during interphase, a large fraction of MN collapses. In this context, ruptures of the membrane are not repaired and trigger MN disruption followed by massive DNA damage and genomic instability [93]. Recently, Vietri and coworkers have elegantly linked this process, known as catastrophic nuclear envelope collapse, to the MN inability of limiting CHMP7-LEMD2 accumulation to the site of lesions. The role of this complex in recruiting ESCRT-III/VP4 to the site of nuclear envelope ruptures is discussed in more detail in Section 3.4.2. As a consequence, ESCRT-III over-accumulates, causing dramatic membrane distortion, DNA stress, and, in the end, chromosome fragmentation [94]. This work, on one hand, further supports the notion that ESCRT-III nucleation and polymerization need to be finely regulated in physiological processes to keep cells healthy and alive. On the other hand, it clearly indicates a role for ESCRT-III in genome instability and in tumorigenesis. Interestingly, different ESCRT components have been linked to cancer development by mechanisms other than defective cytokinesis. Among these mechanisms, which have been extensively reviewed elsewhere [95,96,97], is the control of downregulation of tumor-related receptors by their correct sorting into MVBs, as well as some of the functions displayed by ESCRT proteins in the nucleus that are discussed later in this review.

### 3.4. Involvement of the ESCRT Machinery in Damage Repair of Cellular Membranes

#### 3.4.1. Plasma Membrane Repair

Plasma membrane damages can occur in response to different pathophysiological insults, pathogen-mediated ones included. These lesions compromise cell life, thus need to be rapidly repaired. Recruitment of lysosomes to the plasma membrane triggered by Ca^2+^ influxes is one of the main repair mechanisms for lesions ranging from 200 to 500 nm in size [98,99]. ESCRT machinery, by contrast, appears to be involved in repairing smaller lesions (<100 nm) of the cell surface. In this case, Ca^2+^ influx determines a rapid localization of CHMP4B to the sites of damages. Additional ESCRT-III components along with VPS4 are then recruited to the sites of the lesions, although CHMP4B seems to play a major role in injury resealing [99,100,101]. Interestingly, Alix and TSG101 are the main ESCRT-III assembly factors in this context, while ESCRT-0 and ESCRT-II, as well as additional known assembly cofactors (CHMP6), seem to be dispensable [101]. In particular, Alix interacts with the plasma membrane where it binds TSG101 in a Ca2+ dependent manner [102]. Thus, ESCRT-III plays a crucial protective role in contrasting membrane lesion-mediated types of cell death [103,104]. As an example, it has been shown that ESCRT-III can rescue cells from early-stages of necroptosis, at least temporarily, by acting on the membrane microdomains that are permeabilized by mixed lineage kinase domain-like pseudokinase (MLKL), one of the necroptotic proteins functioning at the plasma membrane [104].

#### 3.4.2. Nuclear Envelope Maintenance and Repair

ESCRT-III is a key player also in the safeguard of nuclear membrane integrity. The nuclear envelope is responsible for the compartmentalization of the cell genome and, thus, of all the nuclear activities. Therefore, its rupture is associated with diseases [105]. At the structural level, the nuclear envelope is a double membrane encompassing the INM and the outer nuclear membrane (ONM), as well as nuclear pore complexes (NPCs) that are involved in controlling the trafficking of macromolecules in and out of the nucleus. Nuclear envelope ruptures can be either a physiological or a pathological event. Indeed, during a normal cell cycle, the nuclear membrane breaks down to allow the interaction between the mitotic spindle and chromosomes. Next, the nuclear envelope reassembles to form the nuclei of the daughter cells [106]. ESCRT-III and VPS4 are involved in the late steps of the reassembly process by executing membrane fission required for sealing the nuclear envelope [107,108]. As seen in the cytokinesis abscission, ESCRT-III recruits the AAA-ATPase spastin to accomplish microtubule severing, a step that is crucial for nuclear compartmentalization [107,108,109]. Interestingly, in this case, recruitment of ESCRT-III does not occur through the canonical ESCRT targeting/bridging factors. By contrast, it is mediated by CHMP7, an accessory ESCRT-III protein [107] that works in concert with the nuclear envelope protein LEMD2 and with additional regulator factors [109,110]. This fundamental function of ESCRT-III appears to be evolutionarily conserved, thus further supporting the notion that ESCRT-III is the central complex of ESCRT machinery [111]. In addition to the involvement in nuclear membrane reconstitution upon mitosis, ESCRT-III core subunits and VPS4 have been described to play a role in sealing the usually transient nuclear envelope damages that occur during the interphase and under pathological conditions [105,112,113,114]. Interestingly it has been recently reported that also the barrier to autoantigen factor (BAF) functions in nuclear envelope repair, most likely upstream of ESCRT-III [115]. Thus, BAF may be involved in an alternative pathway to maintain/reconstitute nucleocytoplasmic compartmentalization in cells lacking ESCRT-III. Finally, ESCRT-III appears to act also in the NPC quality surveillance [116].

#### 3.4.3. Endolysosomal Membrane Repair

Injuries in endolysosomal membranes can be caused by different chemical and biological stresses, as well as by pathogens. Recent evidence indicates that damages in these membranes lead to the Ca^2+^ and ESCRT-I-mediated recruitment of ESCRT-III. By contrast, ESCRT-0 is not required [117,118]. Interestingly, both TSG101 and Alix are engaged by damaged lysosomal membranes and seem to cooperate in early events after injuries [117,118,119]. Data suggest that ESCRT-III represents the first line of intervention in lysosome repair. Indeed, it does not require large ruptures to be activated in its sealing function [117]. The role played by the ESCRT machinery in the repair of lysosome damages has been better analyzed in the context of injuries caused by pathogens, e.g., intracellular bacteria that exploit phagosomes for their survival. For instance, it has been reported that ESCRT-III is localized to the phagolysosomes that contain replicating Coxiella burnetii, most likely to repair membrane lesions induced by bacterial factors/proliferation [120]. Importantly, replication of the bacterium is impaired by TSG101 depletion [118], indicating that ESCRT machinery is crucial to maintain the integrity of the bacterium-colonized phagolysosomes and, thus, to sustain bacterial replication. ESCRT-III is also engaged by Mycobacterium tuberculosis-damaged phagolysosomes. In this way, bacterial virulence factors are prevented from being released in the cytosol [121]. Overall, these findings point to the ESCRT machinery as a potential target for antimicrobial drug design.

In conclusion, ESCRT machinery is involved in many fundamental cellular processes, and when it is not correctly working or it is not finely regulated, it is linked to diseases, cancer included [97]. The importance of ESCRT machinery is further highlighted by the result of knockout studies carried on in mice. Indeed, the loss of TSG101, VPS25, and CHMP5, components of ESCRT-I, ESCRT-II, and ESCRT-III, respectively, is embryonically lethal in mice [122,123,124]. By contrast, deletions of core ESCRT genes in the yeast Saccharomyces cerevisiae are not lethal and enabled the study of ESCRTs in MVB biogenesis. In addition to yeast, Drosophila [125,126] and Arabidopsis thaliana [127] have also been employed as models to analyze the effects of the removal of specific ESCRTs on different cellular pathways. Finally, works based on strategies (mainly small interfering RNAs and more recently CRISPR-Cas9 editing) aimed at silencing the expression of ESCRT factors/cofactors in mammalian cells have been crucial to elucidate the key players in each process and their interplay. Some of these studies, focused on the main cellular processes addressed in this review, are summarized in Table 1.

## 4. ESCRT Machinery and Viral Replication Cycle

Being obligate intracellular parasites, viruses have evolved to hijack fundamental and highly conserved cellular pathways/machineries to execute each step of their replication. This concept is true without any exception, and it is a feature shared among viruses infecting both prokaryotic and eukaryotic cells. Taking into account the crucial functions played by ESCRT proteins, and especially by ESCRT-III and VPS4, in cell life, it does not come as a surprise that viruses can interfere with ESCRT-mediated processes or directly exploit the ESCRT machinery to maximize their chances to establish a successful infection in the host. The main steps of viral replication involving the entire ESCRT machinery or some selected components are reported in Figure 2.

First, viruses have evolved to either antagonize or take advantage of ESCRT-driven processes as autophagy and EV secretion. The interplay between autophagy and viruses has been excellently reviewed elsewhere [132]. We mention the involvement of autophagy in viral transmission later. As far as EVs are concerned, it is interesting to note that they can act both as promoters or inhibitors of viral spreading and infection [8,133]. This dual effect is well exemplified by EVs released from human immunodeficiency virus type 1 (HIV-1)-infected cells. On one hand, EVs can be loaded with HIV proteins that, once transferred to neighboring cells, increase their susceptibility to viral infection. Among these proteins, the accessory Nef can be recruited into EVs [134] and, upon delivery to latently infected cells, it can activate viral replication [135]. Furthermore, Nef-containing EVs have been described to exert several effects on CD4^+^ T cells. For instance, it has been reported to induce senescence and death or to suppress their cytotoxic activity [136,137]. At the same time, Nef-positive EVs affect humoral immune response by inhibiting the production of IgA and IgG by B-lymphocytes [138]. Interestingly, it has been reported that EVs released from HIV-1 infected cells are capable of transferring viral co-receptors to recipient cells, thus increasing their susceptibility to viral infection [139,140]. Finally, EVs have also been found to carry the HIV transactivation response element (TAR) [141]. TAR is a stem-loop-shaped sequence present at the 5′ end of HIV transcripts that interacts with Tat, the main viral transactivator protein, and upregulates viral RNA production. The TAR molecule delivered by EVs could be processed into miRNAs that target a Bcl-2 interacting protein, resulting in a block of apoptosis and thus supporting virus production [141]. On the other hand, it has been shown that EVs produced by infected cells can contain APOBEC3G, one of the main host anti-HIV proteins, while the viral protein Vif, which antagonizes APOBEC3G, is not recruited into EVs [142]. APOBEC3G-loaded EVs would then render recipient cells more resistant to infection. Another example of EVs carrying anti-HIV factors is the vesicles containing the antiviral soluble host molecule cGAMP that acts through interferon and innate immune responses [143]. In conclusion, the interplay between EVs and HIV appears to be extremely complex and not fully disclosed yet [144]. Additional examples of viruses adopting exosomes to facilitate their spreading are the hepatitis B virus [145], HTLV-1 [146] as well as rhinovirus. In the latter case, virus infected cells secrete EVs that induce the upregulation of viral receptors in monocytes, thus increasing the spectrum of cells that can be infected by the virus [147]. Interestingly, it has been recently reported that also cells infected with viruses belonging to the Coronaviridae family, coronaviruses, may produce exosomes with different functions in the replication cycle, pathogenesis and cell response to infection [148]. A coronavirus, the severe acute respiratory syndrome-coronavirus-2 (SARS-CoV-2), is the virus causing the current pandemic. In this context, it has been shown that exosomes can transfer the angiotensin-converting enzyme 2 (ACE2), the main receptor that allows SARS-CoV-2 entry, to other cells rendering them susceptible to viral infection [149]. However, the same EVs might also work as a decoy for circulating SARS-CoV-2 [150], thus suggesting a potential role as therapeutic agents. The idea of using exosomes to interfere with viral infections is not novel [151], as well as the possibility to develop vaccination strategies based on EVs loaded with viral antigens/nucleic acids to stimulate antiviral immune responses, as it naturally occurs from infected cells. Finally, it must be mentioned that EVs can also transfer different miRNAs and even long non-coding RNAs that can affect viral spreading/pathogenesis as well as the host immune system [152,153,154]. The role of EVs in viral transmission is further discussed in Section 4.4 below.

**Figure 3:**
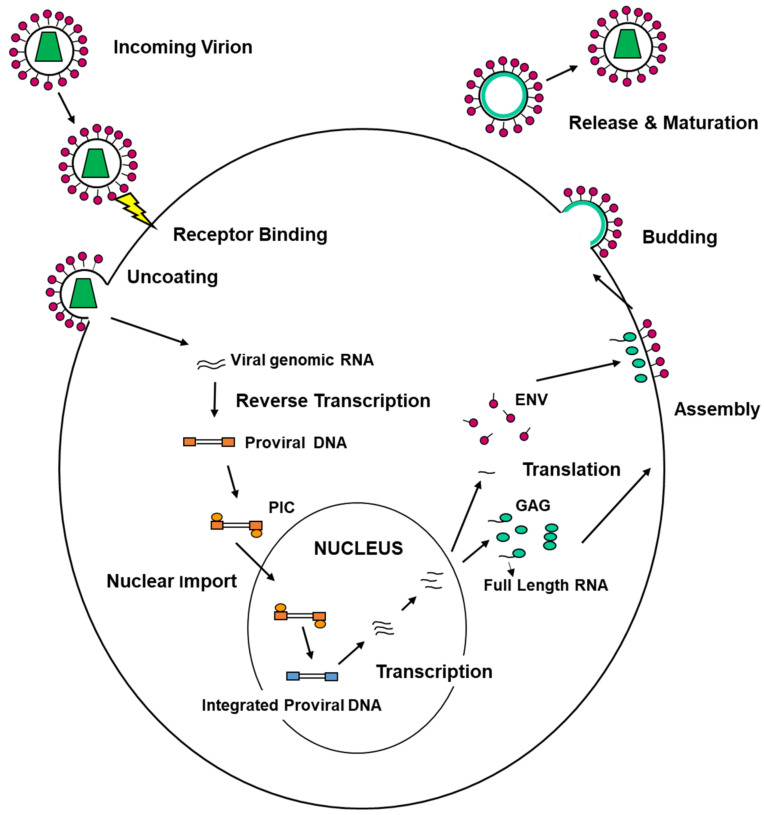
Schematic representation of the main steps in a typical viral life cycle. The figure reports the replication of HIV-1. Upon entry into target cells, mediated by fusion of the viral envelope with the cell surface, the viral genome, which is single-stranded positive RNA (two molecules per viral particle), is partially uncoated from the virion structural proteins, although it is shielded by viral and cellular factors, not entirely known yet (pre-integration complex, PIC). PIC travels to the nucleus and, at the same time, the viral RNA is retrotranscribed by one of the viral enzymes into double-stranded DNA (provirus). Once entered the nucleus, the proviral DNA is integrated into the cellular genome and becomes part of it. Indeed, it is the cellular RNA polymerase II that transcribes the integrated provirus in mRNAs that are translated into the cytosol to give rise to the structural proteins Gag-Pol and Env. The first one drives—in concert with cellular proteins—the assembly and egress of the progeny virions. The latter enriches the viral envelope and is essential to bind the cellular receptors during the next replication round. Regulatory and accessory proteins are also translated. Furthermore, full-length genomic RNAs are also produced to be packaged into the novel budding particles. Once released in the extracellular environment, the new virions terminate their maturation, and they become infectious. Different viruses, due to the specific biological features (in a particular type of genome and presence or absence of an envelope), might differently execute the steps of their life cycle. However, the main steps remain the same: entry, uncoating, genome replication and transcription, protein translation, assembly of progeny virions, egress from infected cells. These stages may present certain differences in the different viral families, also due to the different biological features that characterize each family itself (with the type of genomic nucleic acids and presence or absence of a lipid envelope enwrapping the particle as the main ones in this context). What remains a common trait is the necessity for the virus to interact with the cellular apparatus throughout its replication. This is also the main reason why viruses can alter cell functions, thus causing a disease, and are all pathogenic.

On the other hand, viruses are able to exploit the ESCRT machinery to execute their life cycle. Viral replication encompasses different steps that can be summarized as follows: entry into target cells, uncoating of the viral genome, replication and transcription of the viral genome, assembly of new viral progeny, egress from infected cells (Figure 3).

Seminal studies aimed at dissecting the mechanisms allowing the egress of HIV-1 from infected cells discovered the role played by the ESCRT machinery in the viral life cycle. Some of these studies have also contributed, over the years, to our understanding of the functioning of the machinery in fundamental cellular processes, despite its involvement in viral replication. It is now clear that different viruses can exploit ESCRT proteins or part of them to accomplish virtually all phases of their replication cycle. Interestingly, the interaction between ESCRTs and viruses is of ancient origin, as it has been shown that in Archaea, ESCRT-III components and VPS4 homologs support the replication of the Sulfolobus-turreted icosahedral virus [2]. We summarize below some of the functions of the ESCRT machinery in the viral life cycle with a special accent on the late steps of HSV-1 replication.

Finally, supporting a pivotal role of the ESCRT machinery in the viral life cycle, the host immune system evolved to target this pathway to control infections. In this context, the interferon-stimulated gene 15 (ISG15) represents a key player as it targets the ESCRT proteins in budding complexes to block the release of viruses [155,156]. Furthermore, specific polymorphisms within the 5’ sequence of TSG101 encoding gene (between the −183 and +181 nucleotides with respect to the translation start codon) have been associated with the acquired immunodeficiency syndrome (AIDS) progression, likely due to an effect on plasma HIV-1 load [157]. Overall, these findings strongly suggest that ESCRT proteins and TSG101, in particular, may be interesting targets for the development of antiviral drugs. In this context, pump inhibitors of the prazole family, which interact with TSG101, have been already tested and proved to efficiently inhibit a number of enveloped viruses [158,159], as we further discuss later.

### 4.1. ESCRT Machinery and Viral Entry

To enter into animal cells, viruses must cross the plasma membrane. This non-trivial process can be executed by two main routes: (1) fusion of the viral envelope (if present) with the cellular membrane, a strategy that does not cause membrane injuries [160]; (2) receptor-mediated endocytosis of viral particles that occurs for both enveloped and naked viruses. Members belonging to Picornaviridae, a family encompassing small naked RNA viruses, in most cases enter host cells by creating a pore within the endosomal membrane to translocate their genome in the cytosol where it is replicated [161]. Viruses that exploit the endolysosomal pathway need to escape from it to gain access to the cytosol before undergoing degradation or recycling back to the plasma membrane [162]. In the case of enveloped viruses, this step is carried out by fusion of their envelope with the endosomal membrane, a process that, as mentioned above, should not determine ruptures of the phospholipid bilayer. On the other hand, naked viruses usually cause lesions while crossing the endosomal membranes. This process would result in the exposure of the organelle content in the cytosol with toxic effects and triggering of inflammation. Although most of the mechanisms facilitating this step and the network of cell/viral proteins playing a role are still to be fully elucidated, evidence indicates that ESCRT machinery may play a role in this context, especially when small injuries are involved. It must be mentioned that, at least to our knowledge, this aspect has not been investigated yet. However, it is interesting to note that several viruses display, within virion structural proteins, conserved and short proline-rich motifs, known as late domains (L-domains) that are better described later and that interact with ESCRT and ESCRT related factors [163]. These domains, when exposed in entering viral particles, could act to recruit the ESCRT machinery, for instance, to control membrane damage. Interestingly one of these L-domains, the PPxY motif, is present in the adenoviral membrane lytic protein VI [164]. Adenoviruses are naked DNA viruses that have been extensively studied also with respect to the mechanism of entry into target cells. Upon receptor-mediated endocytosis, adenoviral particles undergo capsid perturbation with the release of the protein VI. Protein VI displays membrane lytic activity [165], and it is crucial for viral infectivity [166]. Membrane damages create pores large enough for enabling viral particle access to the cytosol [167,168]. Protein VI is also known to counteract cellular autophagy, which is activated to remove damaged endosomes [169]. Currently, it is not clear whether this process relies on the ESCRT machinery, although protein VI can interact through a PPxY motif with the Nedd4 ubiquitin ligase, one of the E3 enzymes playing a role in certain ESCRT-mediated processes, as better detailed below. It would be of importance to investigate whether protein VI can also engage the ESCRT machinery prior to exerting its control on autophagy by preventing the cell from repairing small endosomal lesions. Indeed, this could represent a novel and additional mechanism of cell-intrinsic response to a viral infection that may even go beyond the adenoviral entry.

As additional hints on the role that ESCRT components play in viral entry, it has been demonstrated that Kaposi’s sarcoma herpesvirus (KHSV) hijacks the ESCRT machinery for efficient entry in endothelial cells [170]. HRS and TSG101 are key factors in this context. Interestingly, the involvement of TSG101 is common among viruses besides KHSV that enter target cells by micropinocytosis. Indeed, this ESCRT-I component has been shown to contribute to the entry of the Crimean-Congo hemorrhagic fever virus (CCHFV), human papillomaviruses, and echovirus-1 [171,172,173]. Furthermore, in the case of KHSV, TSG101 is also important for the correct trafficking of viral particles through the endosomal pathway [170]. In the case of CCHFV, a tick-borne bunyavirus that can cause a severe clinical manifestation in humans, not only silencing of TSG101 but also depletion of Vps24, VPS4 and Alix inhibits infection. Moreover, virions are trapped in the MVBs when cells are treated with bafilomycin A. This finding suggests that these organelles are the site of virus-endosome membrane fusion. In addition, the highly pathogenic Old World arenavirus Lassa virus (LASV) and the prototypic arenavirus lymphocytic choriomeningitis virus (LCMV) have been described to exploit HRS, Tsg101, ESCRT-III factors, as well as VPS4 to enter into cells [174]. Finally, a study adopting a genome-wide RNA-interference screening has suggested a role for ESCRT components necessary for ILV formation in rotavirus cell entry [175]

### 4.2. ESCRT Machinery Involvement in the Formation of Viral Replication/Assembly Compartments

Once they get access to the cell, several RNA and DNA viruses remodel cellular membranes and exploit host proteins to build specialized compartments where viral genome replication takes place. In these virus-induced “factories”, viral and cellular factors involved in the process are concentrated and preserved from innate immune response mechanisms. Replication compartments originate from different cell membranes, including the plasma membrane, ER, GA, peroxisomes, mitochondria and endosomes [7]. Their biogenesis resembles the ILVs formation process. However, in this case, a membrane fission event does not take place. In addition to genome replication, these compartments can also facilitate the assembly of viral progeny. Working as a shield towards antiviral defenses, the replication compartments are especially useful for most RNA viruses. Indeed, during replication of their genome, high copies of double-stranded RNAs are produced that are the main trigger of the interferon response. Interestingly, ESCRT components have been shown to play a role in the formation of replication compartments of different plus-stranded RNA plant viruses [176,177,178,179]. Flaviviridae, a family of single-stranded positive-sense RNA viruses that include some human pathogens as dengue virus (DENV) and Japanese encephalitis virus (JEV), induce remodeling of the ER membranes to form compartments for genome replication and assembly of viral particles. An elegant study performed by adopting a mass spectrometry approach has shown that several ESCRT factors are re-localized to these compartments. In agreement with the functional relevance of this finding, RNAi screening production of both DENV and JEV particles was significantly affected by lack of TSG101 or of specific ESCRT-III components. However, it was unaffected by depletion of VPS4, thus suggesting that a unique and specific set of ESCRT factors contributes to the biogenesis of the flavivirus-induced replication compartment [180]. It must be mentioned that, while in the case of plant viruses, the role of the ESCRT-machinery is clearly linked to the formation of the replication compartments, in the case of other viruses, flaviviruses included, it is very difficult to distinguish between a role in this process or purely in particle assembly. This is particularly true for certain large DNA viruses, as the ones belonging to the Herpesviridae family, the herpesviruses, whose genome replication and particle assembly occur in the nucleus and in the cytosol, respectively. For instance, both VPS4 and CHMP1 have been reported to localize in the proximity of the human cytomegalovirus (HCMV)-induced cytoplasmic assembly compartments [181]. However, it is very difficult to understand whether these proteins participate in the biogenesis of these specialized sites of viral maturation, to the process of viral morphogenesis or to both.

### 4.3. ESCRT Machinery and Viral Egress from Infected Cells

Enveloped viruses are enwrapped by host membranes and usually egress from infected cells without necessarily causing cell death. Examples of enveloped viruses are HIV, herpesviruses, influenza virus, flaviviruses and coronaviruses (SARS-CoV-2 included). Naked viruses are not surrounded by host membranes and typically are released upon cell lysis [65,182]. Examples of un-enveloped viruses are adenovirus, poliovirus, norovirus, HAV. Enveloped viruses leave the cells through a complex process known as budding that requires two main membrane modifications, (i) a deformation around the assembling virions and (ii) fission, resulting in the detachment of the viral particles from the cellular surface. Budding takes place not only at the plasma membrane but also into intracellular organelles that then fuse with the plasma membrane, releasing viral particles in the external environment [183]. Assembly of virions and budding is usually strictly linked processes. As mentioned above, studies aimed at the dissection of the molecular mechanisms enabling HIV-1 budding have been instrumental for understanding how enveloped viruses are released from infected cells and for unrevealing the viral/cellular protein network involved in the process. In 1991, Göttlinger and coworkers identified in the C-terminal p6 domain of HIV-1 protein Gag, which encodes for the main structural components of the virion, i.e., matrix, nucleocapsid and capsid, a highly conserved PT/SAP motif playing a crucial role in the detachment of budded virions from the cell surface [184]. Starting from this seminal finding, additional research has led to the identification in the Gag protein of all known retroviruses of short proline-rich sequences, named late assembly or L-domains [185], functionally equivalent to the HIV-1 PT/SAP motif. To date, in addition to the PT/SAP motif, typical of most lentiviruses, two further L-domains have been well characterized: the PPXY-type L-domain present in the Gag proteins of oncoretroviruses, first, described in [185], and the YPXnL-type motif, identified in the Gag protein of the equine infectious anemia virus (EIAV) [186]. Besides retroviruses, L-domains and L-domain-like sequences have been identified in the structural proteins of most RNA-enveloped viruses, such as rhabdoviruses, filoviruses, arenaviruses, and paramyxoviruses [186], and in some DNA-enveloped viruses [187,188,189,190,191]. Interesting exceptions have been reported. For instance, neither the hepatitis C virus nor the influenza virus appears to have canonical L-domains or L-domain-like sequences [192,193], while the Bluetongue virus (BTV) presents both a PSAP and a PPXY motif, but at the level of the nonstructural protein NS3 [194]. Early studies indicated that L-domains act independently from their position in the viral protein and can be exchanged between unrelated viruses without losing their ability to mediate budding [195,196,197]. Furthermore, different types of L-domains often occur in combination; for instance, the HIV-1 p6 contains, in addition to its primary PTAP-type L-domain, an auxiliary L-domain of the YPXnL type [20,197,198], and the Ebola virus has a PTAPPEY domain which combines the PTAP and the PPXY types of L-domains [199]. Overall, these features suggested that the L-domains may represent docking sites for a set of cellular proteins belonging to a specific pathway exploited by retroviruses to execute budding efficiently. The first indication on this pathway came from previous observations that cellular proteins, such as the epithelial Na^+^ channel, contained sequences overlapping with the L-domains and that their endocytosis was dependent on these motifs [199,200] and on ubiquitylation [201]. At the same time, studies were supporting the hypothesis on the role of ubiquitin in the viral budding process [50]. In particular, we were able to show that ubiquitin residues involved in retroviral budding were the ones known to play a role in endocytosis [202]. Importantly, in 2001, the Carter and Sundquist laboratories discovered that the PT/SAP motif of HIV-1 p6 binds to TSG101 and that this interaction is crucial for viral budding [203,204]. Later, we show that Alix also plays a role in this context, and it is recruited to the site of budding through a YPXnL domain mapping in the Gag-p6 [20]. Finally, the PPXY type of L-domains was found to bind Nedd4 like ubiquitin ligases that facilitate ESCRT-machinery engagement [50,205]. Over the last two decades, several studies aimed at dissecting the complex viral/cellular protein network that enables most of the RNA-enveloped viruses to hijack the ESCRT-machinery. An updated review on this topic focused on retroviruses was recently published by Rose and coworkers [206], and several excellent and more general ones are available [12,18]. Thus, we do not further describe these aspects here. However, taking inspiration mostly from studies focused on retroviruses, we would like to point out some clear common traits among mechanisms adopted by enveloped viruses to usurp ESCRT-machinery during egress from infected cells: (1) ESCRT-III and VPS4 represent the master players in this process; (2) early data suggested that, while Alix and ESCRT-I were sufficient to recruit ESCRT-III to the side of HIV-1 budding, ESCRT-II was dispensable. By contrast, ESCRT-II is crucial for the exit of the avian sarcoma leukemia virus (ASLV). ASLV Gag displays a PPxY type L-domain [207], while, as already mentioned, HIV-1 Gag contains both a PT/SAP and a YPXnL L-domain. Overall, these findings support the notion that different L domains connect retroviral structural proteins with a different array of ESCRT proteins to execute budding; (3) on the other hand, it has been shown that mutations affecting Alix engagement do not entirely inhibit HIV-1 budding, suggesting a compensatory role for ESCRT-II [208,209] or for additional BRO1 containing proteins in this process. Importantly, most retroviruses have evolved redundant mechanisms to hijack ESCRT-III/VPS4 to the site of budding, as exemplified by HIV [210], and as we reported, for instance, in the case of the closely related feline immunodeficiency virus (FIV) [211]. Strikingly, budding of HIV-1 and FIV devoid of functional L-domains can be efficiently rescued by overexpression of Nedd4-like ubiquitin ligases, although both lentiviruses lack a PPxY motif within their Gag [212,213,214,215]. Finally, retroviruses have been shown to engage ESCRT components also through Gag ubiquitylation [50,204,216], as well as through the Gag Nucleocapsid region [53,211,217,218,219]; (4) RNA-enveloped viruses that seem to egress independently from the ESCRT-machinery, e.g., the influenza virus [220], encode proteins that might functionally replace ESCRT-III [193]; (5) large DNA-enveloped viruses can adopt ESCRT factors to egress from infected cells, but with some clear peculiarities that are discussed later in the case of HSV-1.

### 4.4. EVs and Autophagy in Viral Transmission

As the healthy counterpart, virus infected cells can secrete EVs that can exert different functions as described above. These EVs can shed viral proteins, and nucleic acids and either trigger immune responses or affect recipient cells, favoring viral spreading [8]. Interestingly, EVs and enveloped viral particles share common biogenesis machinery, and in some cases, it is difficult to isolate EVs containing viral molecules from forms of defective viral particles as they display similar density, size, and composition [8]. As mentioned above, viruses are distinguished in enveloped and naked viruses. However, in 2013, this concept was challenged by the finding of both types of particles in the supernatant of liver cells infected with the hepatitis A virus (HAV), as well as in vivo [221,222]. A follow-up study demonstrated that HAV enveloped particles could be found inside MVBs and ESCRT depletion inhibited their release from infected cells, thus suggesting exosomes as an egress pathway. Furthermore, these findings also indicate that the envelopment of HAV relies on the same cellular machinery exploited by most of the known enveloped viruses [65]. Importantly, HAV-containing exosomes, named “quasi-enveloped” HAV (eHAV), are able to protect viral particles from antibody-mediated neutralization. In addition to HAV, also hepatitis E virus (HEV), another naked virus, could be found in MVB. The release of eHEV particles is likely to occur via the exosomal pathway [223,224].

Interestingly, exosomes seem to play a role also in the egress of HIV. Indeed, Gould and coworkers demonstrated that EVs secreted by dendritic cells infected with HIV were able to transfer the infectious virus to CD4+ T lymphocytes [225]. Similar results were reported for hepatitis C virus (HCV). Exosomes loaded with HCV were indeed isolated from infected liver cell lines [226]. In addition, hepatic exosomes allowed transmission of infectious HCV particles in vitro and were resistant to antibody neutralization, at least partially. In agreement, HCV release was found to follow both an exosome-driven and an exosome-free route, as in the case of HAV. Importantly, exosome-associated HCV particles were infectious and resistant to neutralizing antibodies [227]. Furthermore, it was also demonstrated that EVs could deliver HCV replication-competent subgenomic RNAs allowing infection of recipient cells, even in the absence of infectious particles and independently from known viral receptors [228]. This latter aspect is particularly relevant as it would shield the virus from neutralization, an additional immune evasion strategy that may be exploited by other viruses. In this respect, also EVs secreted from Zika virus (ZIKV) or foot and mouth disease virus (FMDV) infected cells contain viral RNAs and proteins and can support viral transmission as well as function as an escape strategy from neutralization [229,230]. Furthermore, infectious particles of severe fever with thrombocytopenia syndrome (SFTS) virus, a tick-borne bunyavirus associated with hemorrhagic fever, were found in exosomes and were shown to infect cells, bypassing the need of classical viral receptors [231]. Finally, DENV-infected insect cells produce EVs loaded with virus-like particles that are able to infect other cells [232].

EVs can contribute to the spreading of infection also by increasing the spectrum of cells susceptible to viral entry and/or viral infectivity. One of the mechanisms allowing this EV-mediated activity is the delivery of viral receptors from the producing cell to the recipient one, as described above. Moreover, in the case of HIV-1, it has been shown that resting CD4+ T lymphocytes, which are usually refractory to infection, become permissive to viral replication by the combined function of the TNF-converting enzyme ADAM17 and Nef, delivered by exosomes [136]. Additionally, Nef-containing EVs can alter lipid rafts on the cell surface, facilitating HIV entry by fusion and thus increasing infectivity [233]. It must be noticed that certain viruses that acquire their envelope by budding into cellular organelles, e.g., the HSV-1, can hijack the exosomes to facilitate their transmission, at least from certain cell types [234,235]. We discuss HSV-1 egress from infected cells later in this review.

Surprisingly, studies carried on with cells infected by certain members of the Picornaviridae family, e.g., poliovirus, revealed that also these viruses could egress from host cells in vesicles before cell lysis [236]. However, these vesicles did not derive from MVBs, but rather from autophagosomes [236,237]. As already mentioned, autophagosomes by fusing with lysosomes display a pivotal role in different aspects of cell biology. However, it is known that these structures can also fuse with the plasma membrane to release molecules and organelles in the extracellular environment, a process known as “secretory autophagy” [237]. Thus, the above-mentioned viruses hijack this specialized type of autophagy to egress from infected cells. Indeed, virus-containing autophagosomes, which in the case of the poliovirus originate from the ER, do not fuse with lysosomes but with the plasma membrane and release vesicles enwrapped in a single membrane and loaded with viral particles [236]. By contrast, to exosome-mediated uptake of viruses, autophagosome-derived vesicles drive viral entry into recipient cells via the respective physiological receptors. This finding suggests that vesicles are disrupted upon binding with the new host cell, releasing particles that need to bind to the surface receptors [236]. Furthermore, lipid recognition seems to play a role as well. Indeed, proteins able to bind the phosphatidylserine, a lipid enriching the autophagosome-derived vesicles, inhibited viral infection [236].

Overall, these findings indicate that vesicles can contribute to cell egress and transmission of both naked and enveloped viruses. The emerging picture is complex, and the impacts on the host immune response and viral pathogenicity urge further studies.

## 5. The Travel of Herpes Simplex Virus Type 1 from the Nucleus to the Extracellular Environment: Is There a Role for the ESCRT Machinery?

The Herpesviridae are large, enveloped DNA viruses that not only replicate their genome within the nucleus, but also start the process of assembling progeny particles in the same cellular compartment. Newly assembled nucleocapsids need to cross the nuclear membranes to translocate into the cytoplasm. In the cytosol, nucleocapsids are enwrapped in proteins that constitute what is known as tegument, an amorphous coat that is typical of herpes viral particles. Next, tegument surrounded nucleocapsids bud into vesicles derived from host organelles to acquire their envelope. Finally, these vesicles fuse with the plasma membrane to release the mature viral progeny in the extracellular environment. Therefore, the biogenesis of herpesviral particles is a multistep process involving at least two budding and fusion events and complex traffic of nascent virions from inside the nucleus to outside the cell. Notably, ESCRT components play a role along this complex process, although with peculiar and distinct features with respect to the better-clarified strategies evolved by retroviruses and several other families of enveloped RNA viruses to hijack the ESCRT machinery during budding. One of the most striking peculiarities in the assembly and maturation of herpesviral particles is the first budding from the nuclear envelope. If ESCRTs are involved in this process, they need to be available within the nuclear environment. This is indeed the case. When TSG101 was described as a key component of the cellular machinery involved in HIV-1 budding, this finding came as a surprise as this factor was mainly known for its nuclear functions, as also suggested by its name (tumor suppressor gene 101). Indeed, TSG101 was initially characterized for its ability to inhibit not only the transcriptional activity of the nuclear hormone receptor superfamily [238,239], but also for its ability to suppress the activity of certain viral promoters and transcriptional activators [239]. As an example, TSG01 can interact with Rta, a transactivator of EBV lytic genes, and, as a consequence, it can be recruited to the viral promoters [240]. TSG101 improves binding of Rta to these promoters and its transactivating activity. Furthermore, TSG101 is known to play a role in the p53 pathway [241]. Indeed, TSG101 inhibits ubiquitylation and degradation of the mouse double minute 2 homolog (MDM2) protein. MDM2 is an E3 ubiquitin ligase, which targets p53 for degradation by recognizing its N-terminal transactivation domain (TAD). Furthermore, MDM2 can also inhibit p53 transcriptional activation. The effect of TSG101 on MDM2 stability results in p53 downregulation accompanied by a stimulation of the cell cycle [242]. As in the case of TSG101, also ESCRT-II components were first described as nuclear proteins. Indeed, the yeast orthologues of ESCRT-II subunits are named ELL-associated proteins (EAPs). ELL binds to the RNA polymerase II, functioning as an elongation factor. Interestingly, also in rats, ELL interacts with three factors belonging to ESCRT-II (EAP20/Vps25, EAP30/Vps22, and EAP45/hVps36), giving rise to a heterotetrameric complex that enhances transcription elongation, at least in vitro [243,244]. Overall, ESCRT-II appears to interfere with the expression of specific genes by regulating the rate of promoter initiation of transcription. Finally, mammalian ESCRT-III subunits (CHMPs) were initially studied for their functions in the nucleus as suggested by their initial name, i.e. chromatin-modifying proteins, that was then changed into charged multivesicular body proteins upon discovery of their cytoplasmic functions. These two names are currently used interchangeably. In the context of CHMP nuclear activities, several studies focused on CHMP1. This protein is characterized by a predicted bipartite nuclear localization signal, and it is found both in the cytosol and in the nuclear matrix. Interestingly, nuclear CHMP1 appears to be post-translationally modified with respect to the cytosolic counterpart. This modification may contribute to the regulation of CHMP1 compartmentalization or to the reason for a different activity of the protein in the two cellular spaces. In the nucleus, CHMP1 interacts with polycomb-like proteins that, along with the other components of the polycomb group (PcG), enable chromatin condensation and gene silencing during development. Furthermore, it has been suggested that CHMP1 might be involved in chromosome condensation during mitosis [245]. Interestingly, in a screening aimed at the identification of human CHMP interactors, 19 out of 45 novel-fished-out factors were nucleus-related proteins [246]. Finally, several CHMPs take part in nuclear sumoylation pathways. These distinct functions of the nucleoplasmic ESCRT components with respect to the activity displayed by the cytoplasmic counterpart are a striking example of the importance/role of cell compartmentalization.

Interestingly, however, herpesviruses, at least HSV-1 and EBV that have been studied under this respect exploit nucleoplasmic ESCRTs to facilitate nuclear inner membrane remodeling and fission in the first envelopment step, as better explained below. Thus, in this context, nuclear ESCRTs function as their cytoplasmic counterpart. HSV-1 belongs to the Alphaherpesvirinae subfamily of the Herpesviridae, and it is a neurotropic virus ubiquitous in the human population. HSV-1 usually causes localized mucocutaneous lesions in the facial region. Primary infection often occurs after direct interpersonal contact and precedes the retrograde transport of the virus to the sensory nerve ganglia, where it establishes a lifelong latent infection with recurrent infections [247]. In fragile patients, HSV-1 can systemically disseminate and cause fatal infections. In particular, HSV-1 can cause acute encephalitis and severe neonatal infections. The process by which HSV-1 virions exit the nuclear and then the cytoplasmic membranes of infected cells is very complex and not fully elucidated yet. Viral DNA replication and gene transcription take place in the nucleus where, during productive (lytic) infection, nucleocapsids are also assembled. Althoug a sufficient dilatation of nuclear pores to allow egress of viral capsids was described [248], the most widely accepted and supported model postulates that HSV-1 capsids by budding from the INM acquire a lipid envelope, which then fuses with the ONM [249]. A central role is played by the herpesviral nuclear egress complex (NEC) that is composed of viral proteins encoded by the UL31 and UL34 genes. NEC forms a hexagonal lattice on the inner surface of the nuclear membrane, and it is sufficient to induce perinuclear vesicles in uninfected cells expressing alphaherpesviral NEC proteins alone. Furthermore, HSV-1 NEC can mediate the budding of membrane vesicles in the absence of endogenous cellular proteins and ATP [249]. The UL34 protein is likely the real effector, but in the absence of the UL31 protein, it fails to localize to the nucleus, as it lacks a nuclear import signal. It is noteworthy that HSV-1s bearing deletions or dominant-negative mutations within the UL34 gene display highly reduced viral infectious titers with a corresponding accumulation of intranuclear viral capsids; however, some infectious virions can still be recovered. Different mechanisms may be activated in this case, including nuclear pore enlargement (see above) or nuclear envelope breakdown, but the relevance of such alternative pathways under physiological conditions is unclear. NEC-mediated budding is also likely to have a quality control function, selecting mature, DNA-filled C-capsids rather than type A and B capsids [250]. Conversely, budding is probably negatively regulated to reduce the number of capsid-less vesicles, for example, by phosphorylation of UL31 by the US3 viral kinase. Regulation of budding is even more complex and could involve tegument proteins that localize to the nucleus, such as UL36, UL47, UL16, UL11 and UL21, along with nonstructural proteins like UL24 [251] and the neurovirulence factor ICP34.5 [252]. De-envelopment of perinuclear enveloped virions (PEVs) is not as well characterized as envelopment: NEC alone is not able to perform it efficiently, and viral glycoproteins gB and gH play a role in the process; however, both proteins are dispensable for it [249].

It has been recently shown that HSV-1 employs nucleoplasmic ESCRT proteins to bud across the INM. In particular, UL34 can interact with Alix in infected cells, and UL31/UL34 leads to the localization of CHMP4B to the nuclear envelope. At the same time, virions accumulate in the perinuclear space of cells depleted of CHMP4A/B/C or Alix, likely because scission at the INM is inhibited [253]. Overall, these data indicate that HSV-1 NEC via Alix can recruit CHMP4 to the site of budding at the INM. However, a dominant-negative version of VPS4 did not abrogate transit of nascent nucleocapsids to the cytosol [253,254,255,256], suggesting no major nuclear egress defects. On the other hand, it has been demonstrated that NEC alone is sufficient to drive membrane scission, at least in vitro [257]. Furthermore, Alix does not appear to be essential for HSV-1 replication [258]. Thus, CHMP4/VPS4 would be necessary to optimize the rate of HSV-1 budding into the perinuclear space, but not strictly required. On the other hand, HSV-1 infection has been shown to trigger autophagy at the nuclear membranes [259,260], leading to degradation of lamins that constitute a barrier for HSV-1 nuclear egress [260,261]. Under this respect, ESCRT-III/VPS4 could facilitate HSV-1 budding from the INM by functioning in the biogenesis of the autophagosome and thus in laminin destruction [66,262]. Finally, ESCRT-III may be recruited to help the nuclear envelope stressed by HSV-1 budding nucleocapsid to maintain its integrity, although, as described above, CHMP-7 and not Alix is the factor recruiting CHMP4B at the INM during this process [263]. Of note, as discussed below, TSG101 has been recently implicated in HSV-1 ability to cross the nuclear envelope. Interestingly also the NEC of EBV, constituted by BFLF2 and BFRF1 proteins, has been described to interact with ESCRT components during EBV capsid nuclear envelopment [264]. In this case, not only Alix, CHMP4B and VPS4 play a role, but also the ubiquitin ligase Itch/AIP4 that belongs to the Nedd4 family of E3 enzymes. As in the case of retroviral Gag, BFRF1, which interacts with Itch, is ubiquitylated at several lysine residues [265]. Mutations of these residues affect the release of enveloped particles from infected cells, likely due to an impairment of the EBV particle trafficking from the nucleus to the cytosol [265]. Although some viral tegument proteins localize to the nucleus, as mentioned above, most of them are acquired in the cytosol: subsequently, virions undergo secondary envelopment by budding into cytoplasmic membranes. The exact nature of this membrane is still under debate. HSV-1 capsids have been observed enveloping at structures morphologically resembling the trans-Golgi network (TGN) [266]. This finding would be consistent with the lipid composition of the final HSV-1 envelope [267]. An alternative model identifies endocytic tubules deriving from the plasma membrane and containing viral envelope proteins at the site of cytoplasmic envelopment [268]. Following secondary envelopment, HSV-1 hijacks secretory pathways to exit the cell by exocytosis, though at late-stages of infection, some virions are released following cell death and lysis. Among tegument proteins, UL36 and UL37 are crucial for envelopment [269] as they mediate both interactions with cytoskeleton proteins to transport virions towards the Golgi apparatus and with envelope glycoproteins. Other tegument proteins are essential (UL48) or auxiliary (e.g., UL51 and UL7) for secondary envelopment [262]. The precise identity of the compartment in which secondary envelopment takes place is still unknown, though it surely involves post-Golgi membranes, which may include TGN or endosomes, or possibly membranes of heterogeneous origin [262]. Viral glycoproteins are trafficked to the same post-Golgi membranes via different mechanisms. In particular, while some viral surface proteins have clear localization signals, others do not [188]. Knockdown of Rab6A, involved in intracellular trafficking, inhibits viral secondary envelopment [268]. Enveloped virions then are translocated to the plasma membrane by a mechanism, which is associated with proteins directly involved in the secretory pathway, such as Rab GTPases, GAP-43, kinesin-1 and SNAP-2, as well as upstream regulators like protein kinase [247]. Since the discovery that the dominant-negative version of VPS4 (VPS-EQ) affects HSV-1 cytoplasmic envelopment and replication [254], several studies have connected ESCRT machinery also to this step of viral egress from infected cells. For instance, it has been shown that dominant forms of different CHMPs can also inhibit HSV-1 replication [258]. In particular, electron microscopy studies suggested that in cells overexpressing VPS-EQ, partially enveloped nucleocapsids of HSV-1 accumulate in the cytosol as the final step of membrane fission into the lumen of a cytoplasmic organelle did not occur [256]. This phenotype is reminiscent of the one found for HIV-1 in cells expressing the same dominant-negative protein or in the case of mutation of the PT/SAP L-domain [20,184]. Importantly, a number of HSV-1 proteins contain binding motifs for Alix as well as for TSG101 [258]. However, neither the dominant-negative version of these proteins nor their depletion affects HSV-1 yield [258]. Interestingly, it has been reported that proton pump inhibitors of the prazole family, by interfering with TSG101, affect not only the budding of HIV-1 but also of different enveloped viruses, EBV included [158,159], and, more recently, in a pre-print manuscript by Leis and coworkers, of HSV-1 and HSV-2 [270]. It must be noted that the drugs appear to inhibit HSV-1 transit through the nuclear membrane and not the final envelopment in the cytosol. In any case, these studies, on one hand, further connect TSG101 to the egress of different viruses, including HSV-1, from infected cells. On the other hand, they support the possibility of adopting ESCRT proteins as a target for antiviral therapy. Despite the presence of a PPxY domain in HSV-1 UL56 protein, there is no evidence that interaction with Nedd4 is involved in ubiquitin-mediated recruitment of the ESCRT machinery to execute viral envelopment. In agreement, the viral RING finger E3 ubiquitin ligase ICP0 does not seem to play a role in ESCRT engagement.

In conclusion, although TSG101 may play a role as suggested by the prazole-based data, the mechanism adopted by HSV-1 to recruit ESCRT-III to the side of the final envelopment is still under evaluation. Interestingly, different studies have focused on the viral tegument and envelope proteins as potential ESCRT-III recruiting factors/cofactors. Under this respect, we were able to show that glycoprotein B (gB), one of the most highly conserved glycoproteins across the Herpesviridae family, was sorted into MVB membranes [188]. In cells expressing VPS4-EQ, the site of intracellular gB accumulation and maturation was altered, indicating that gB traffic was dependent on a functional MVB pathway. Interestingly, gB was ubiquitylated in both infected and transfected cells. A partial deletion of the gB cytoplasmic tail resulted in a dramatic reduction of ubiquitination, as well as of progeny virus assembly and release to the extracellular compartment. Our data support the view that the sorting of gB to MVB membranes may represent a critical step in HSV envelopment and egress [188]. On the other hand, the major and highly conserved tegument protein UL36 appears to be a very promising candidate due to its essential role in the final envelopment of HSV-1 particles. Supporting this hypothesis, UL36 encompasses an amino-terminal functional deubiquitylase (DUB) domain [271,272] that would fit with the role of ubiquitin in ESCRT-mediated viral budding described above. We were able to show that HSV-1 UL36 regulates the ubiquitination of TSG101 [189]. However, as UL36 DUB activity is not essential for HSV-1 replication [258,273], we could not conclude that this finding is relevant in the context of viral envelopment. Under this respect, an elegant study carried on by adopting light and scanning electron microscopy strongly suggested a role for UL36 in the recruitment of capsids to the site of ESCRT-III/Vps4 localization [256], although this effect may be indirect. Finally, a recent study has demonstrated that the tegument protein UL51 has an impressive structural similarity to the ESCRT-III subunit CHMP4B, and can give rise to long ESCRT-III-like polymers in vitro [274]. Thus, UL51 could facilitate CHMP polymerization or may even functionally replace CHMPs originating ESCRT-III-like filaments. Conceivably, any envelope and tegument proteins involved in cytoplasmic envelopment could facilitate the recruitment of ESCRT components as well as the docking of nucleocapsid-containing vesicles at the assembly sites. Studies may also be complicated by the redundant functions of different viral factors.

Overall, although ESCRT components are clearly involved in HSV-1 final envelopment and egress from infected cells, there are still several open questions to address before fully understanding all the peculiar aspects of these complex late steps of viral replication.

## 6. Conclusions and Outlook

The ESCRT machinery is involved in many fundamental cellular pathways. In eukaryotic cells, ESCRTs contribute to both maintenance of cell compartmentalization, as well as to the biogenesis of vesicles and thus to the crosstalk among organelles and between the cell and the extracellular environment. Over the past years, efforts have been made to gain further insights into the molecular mechanisms enabling most of the ESCRT-mediated pathways. However, there are still unmet questions that need to be addressed, including a better understanding of the precise role of the individual interactions and steps allowing the recruitment of the same set of proteins to different locations to facilitate or play key roles in a variety of processes. Viruses interact with the ESCRT components in different steps of their replication cycle and/or influence ESCRT-mediated actions to their advantage. Thus, the study of viral/ESCRT protein interplay might contribute to unraveling aspects of the ESCRT machinery functions as well as of its regulation that are still under debate. In this context, HSV-1 and herpesviruses, in general, might be of great interest as they exploit ESCRT components in peculiar ways that differ from the ones that have been well characterized for HIV-1 and most RNA-enveloped viruses. Particularly interesting is the fact that HSV-1 uses both nuclear and cytoplasmic ESCRT factors to cross the nuclear and plasma membrane barriers. Furthermore, nascent virions engage cytoskeleton elements as well as secretory pathways to travel throughout the cell and exit in the extracellular environment. Thus, studying the missing links of this travel may contribute to our knowledge not only on the ESCRT machinery functions, but also on the molecular exchanges between intracellular organelles and compartments.

## Figures and Tables

**Table 1:** Table reports the key players for the major processes in mammalian cells involving ESCRTs. The results of silencing studies supporting the role played by these key players are also displayed.

Cellular Process	Key Player/s	Effect of Silencing	References
**MVB Biogenesis**	ESCRT-0 (HRS) ESCRT-I (TSG101) ESCRT-III/VPS4	HRS depletion: enlarged MVBs with few ILVs TSG101 depletion: MVB formation strongly reduced ESCRT-III/Vps24 depletion: smaller MVBs in clusters HRS TSG101, Vps22 and Vps24 co-depletion: MVBs and ILVs still formed	[53,128,129,130]
**Autophagy**	ESCRT-I (VPS37A) ESCRT-III (CHMP2A)/VPS4	VPS37A depletion: accumulation of phagophores due to defects in autophagosome completion CHMP2A depletion: accumulation of immature autophagosomal structures; impairment of autophagic flux; inhibition of phagophore sealing during mitophagy CHMP2A, CHMP3, CHMP7 depletion: increase in immature autophagosomal membranes under starvation CHMP2A, CHMP4B and VPS4 depletion: inhibition of mitophagy	[66,67]
**Cytokinesis**	ESCRT-I (TSG101)/ESCRT-II Alix ESCRT-III (CHMP-6,CHMP4B,CHMP4C)/VPS4	Alix depletion: an increase in multinuclear cells; furrow regression; a failure in CHMP4C recruitment to the midbody; CHMP4B still recruited TSG101 and Alix co-depletion: failure in CHMP4B recruitment to the midbody; multinucleation non aggravated Alix, VPS22, and CHMP6 co-depletion: CHMP4B is not recruited to the intercellular bridge CHMP4C depletion: altered cytokinetic arrest in the presence of chromosomal problems; furrow regression and binucleation	[73,74,80,88,131]
**Cell Membrane Repair**	ESCRT-I (TSG101) Alix ESCRT-III (CHMP4B)/VPS4	Alix, CHMP2B VPS4 depletion: failure of the repairing process followed by cell death (CHMP4B and VPS4 silencing) CHMP2A depletion: impairment of the repairing process CHMP3 depletion: no significant effect	[99,101]
**Nuclear Membrane Repair**	ESCRT-III (CHMP4B, CHMP7)/VPS4	Alix, HD-PTP, HRS, TSG101 depletion: no effects on CHMP4B recruitment to the site of ruptures CHMP7depletion: failure of CHMP4B recruitment to the nuclear envelope	[107,108]
**Lysosomal Membrane Repair**	ESCRT-I (TSG101) Alix ESCRT-III (CHMP2A, CHMP4B)/VPS4	HRS depletion: no effect on CHMP4B recruitment to lysosomes TSG101 depletion: consistent delay in CHMP4B recruitment CHMP2A depletion: increased accumulation of CHMP4B on damaged lysosomes Alix depletion: no detectable effect on CHM4B recruitment TSG101 and Alix co-depletion: almost complete abrogation of CHMP4B recruitment; failure of recovering of damaged lysosomes	[118,119]
